# Efficacy and safety of electroacupuncture in treatment of cervical spondylosis: A protocol of randomized controlled trial

**DOI:** 10.1097/MD.0000000000025570

**Published:** 2021-05-07

**Authors:** YaZhou Zhou, WenGang Wang, Ke Tian, Hui Huang, Mengrui Jia

**Affiliations:** a Henan Medical College, Zhengzhou; b The First Affiliated Hospital of Zhengzhou University, Zhengzhou, Henan province, China.

**Keywords:** cervical spondylotic radiculopathy, electroacupuncture, protocol, randomized controlled trial

## Abstract

**Background:**

Cervical Spondylotic radiculopathy (CSR) is the most common spinal degenerative disease. Its clinical manifestations are pain and numbness in the neck and arm and limitation of neck movement, which greatly affects the life and work of patients. Acupuncture and electroacupuncture are commonly used in China, the efficacy of acupuncture has been confirmed. Existing evidence shows that electroacupuncture seems to be better than acupuncture, but there is a lack of clinical research to directly compare the two.

**Methods:**

This is a prospective randomized controlled trial to compare the efficacy of electroacupuncture and acupuncture in the treatment of CSR and to explore the safety and potential mechanism of electroacupuncture in the treatment of CSR. Approved by the Clinical Research Ethics Committee of our hospital, the patients are randomly divided into an experimental group (electroacupuncture group) or control group (acupuncture group). The patients are followed up for 30 days after 4 weeks of treatment. Observation indexes included VAS score, Neck Disability Index, Yasuhisa Tanaka 20 Score Scale, adverse reactions and so on. Finally, the data will be analyzed by SPSS 18.0 software.

**Discussion:**

This study will directly compare the advantages and disadvantages of electroacupuncture and acupuncture in the treatment of CSR. The results of this study will help to guide patients with CSR to choose appropriate treatment.

**Trial registration:**

OSF Registration number: DOI 10.17605/OSF.IO/9MKPN

## 1 Introdoction

Cervical spondylosis is a very common disease. Cervical Spondylotic radiculopathy (CSR) is one of the most common types, accounting for about 60% to 70% of all cervical spondylosis.^[1]^ Every year, about 1.6 million people around the world suffer from dysfunction, which seriously affects the quality of life.^[2]^ CSR is mainly due to degenerative changes such as protrusion or prolapse of nucleus pulposus, hyperosteogeny of posterior facet joint or bone spur formation of uncinate joint, which cause stimulation and compression to spinal nerve root and appear corresponding symptoms and signs.^[1,3]^ Due to the aging of the population, the change of life style and the increase of work or life pressure, the incidence of CSR is increasing year by year.^[4]^ Its clinical manifestations are mainly neck, arm pain, numbness, limited neck movement, seriously affecting people's life and work.^[5]^

The main purpose of CSR treatment is to relieve pain, numbness and discomfort, improve function and improve quality of life. At present, conservative treatment is still the main treatment of CSR, but the traditional treatment is limited because of its low efficacy.^[6]^ These treatments include non-steroidal anti-inflammatory drugs (NSAIDs),^[7]^ muscle relaxants,^[8]^ epidural steroid injections,^[9]^ physical therapy^[10]^ and so on. With the exploration of complementary and alternative therapies, traditional Chinese medicine intervention methods, such as Chinese herbal medicine,^[11]^ cupping therapy,^[12]^ acupuncture,^[13]^ massage,^[14]^ have been widely used in the treatment of cervical spondylosis, among which acupuncture is the most popular treatment. There has been evidence that acupuncture has significant anti-inflammatory and analgesic effects, and can significantly improve the quality of life of patients with CSR.^[15]^

Electroacupuncture is a therapy which adds electrical stimulation on the basis of acupuncture. It has been widely used in bone and joint diseases, and has been proved to be effective in some cases of failure of traditional acupuncture treatment.^[16]^ It combines the meridian theory of traditional acupuncture and the advantages of low-frequency current, and can set the frequency and intensity of stimulation. Clinical studies have shown that electroacupuncture increases the expression of integrin β 1 and Aktinhibits by inhibiting TNF--TNFR1-caspase-8 signal pathway, and inhibits the apoptosis of AF cells, which have been proved to be a good alternative therapy for cervical spondylosis.^[17]^ Based on the current evidence, it is speculated that the effect of electroacupuncture on CSR may be better than that of traditional acupuncture. However, there is still a lack of clinical research on the direct comparison of the two treatments in the treatment of CSR. Therefore, we intend to use this randomized controlled trial to evaluate the advantages and disadvantages of the two treatments in the treatment of CSR, and to explore the safety and potential mechanism of electroacupuncture in the treatment of CSR.

## 2 Materials and methods

### 2.1 Study design

**Figure 1:**
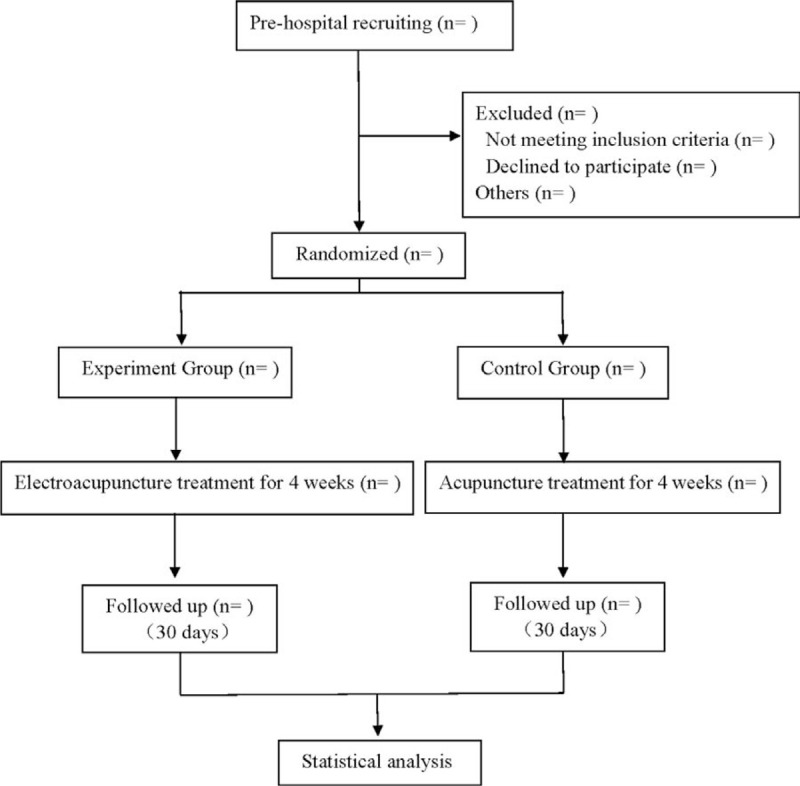
Flow diagram.

This is a prospective randomized controlled trial to compare the efficacy of electroacupuncture and traditional acupuncture in the treatment of CSR, and to study the efficacy and safety of electroacupuncture in the treatment of CSR. This experiment will follow the Standards for Reporting Interventions in Clinical Trials of Acupuncture^[18]^ and the comprehensive trial report standard.^[19]^ The flow chart is shown in Figure 1.

### 2.2 Ethics and registration

This research scheme is in line with the Helsinki Declaration and has been reviewed by the Clinical Research Ethics Committee of our hospital. This lab has been already registered in OSF (registration number: DOI 10.17605/OSF.IO/9MKPN). Before being randomly divided into groups, all patients are required to sign an informed consent form, and they can choose whether or not to continue the trial at any time.

### 2.3 Sample size

The calculation of sample size is based on the mean and standard deviation of the visual analogue scale (VAS)^[20]^ score after treatment. According to the results of the pre-experiment, the experiment group is 2.41 ± 1.49, and the control group is 3.25 ± 1.28. Set α = 0.025, unilateral test, β = 0.10. Calculated by PASS15.0 software, each group needs 59 participants, with an estimated withdrawal rate of 10%, and each group will include 66 participants.

### 2.4 Patients

Inclusion criteria: ① conforms to the diagnosis of cervical spondylotic radiculopathy (diagnosis refers to the diagnosis criteria of cervical spondylopathy issued by Chinese Rehabilitation Medical Association (2010) ^[21]^); symptoms and signs of ② include pain and numbness along the spinal nerve roots with positive foraminal compression and/or brachial plexus pull tests. Imaging (cervical spine X-ray, magnetic resonance imaging or computed tomography) consistent with clinical symptoms; ③ age 18–70 years; ④ not receiving acupuncture or other physical or medical treatment within 7 days; ⑤ signing informed consent form.

Exclusion criteria: ①other types of cervical spondylosis, such as vertebral artery type cervical spondylosis, sympathetic cervical spondylosis, cervical Spondylotic myelopathy, etc.; ②patients with history of neck trauma, neck fracture or operation; ③serious systemic complications such as cardio-cerebrovascular disease, tumor, diabetes, kidney disease or digestive system disease; ④pregnancy or lactation; ⑤refusal or fear of acupuncture.

### 2.5 Randomization and Blinding

Random sequences will be generated by SAS 9.3 software (SAS Institute, Cary, NC, USA) by independent statisticians who do not participate in the implementation of the experiment or statistical analysis. Eligible participants will be randomly assigned to the electroacupuncture group or acupuncture group according to a 1:1 ratio. The clinical research coordinator entered participant information on the tablet and is given a random number. The research assistant gets the allocation of participants from the computer. Throughout the study, the research assistant is responsible for screening, recruiting participants, and assigning random numbers to participants who have been included. The outcome evaluator is responsible for the evaluation of the scale. Considering the actual operational nature of acupuncture, participants and practitioners may be aware of random distribution. However, the evaluators of research results and the statisticians of data statistics and analysis do not know about the distribution.

### 2.6 Intervention measures

Control group: patients take proper posture and fully expose the operation site, and the acupuncture site is locally sterilized with 75% alcohol. The acupuncture points are: Dazhui (GV 14,1 point), Fengchi (GB 20, bilateral), Cervical Jiaji Points (EX-B2, bilateral), Jianjing point (GB 21, bilateral),) using disposable stainless steel acupuncture needle (Suzhou Hualun Medical Appliance Co., Ltd, Suzhou, China). Acupuncture is performed by an acupuncturist with a license of traditional Chinese medicine and at least 3 years of clinical experience. Acupuncture manipulations are stimulated for 10 s to achieve the sense of “getting Qi”. The needles are retained for 30 min, 4 times a week for 4 weeks.

Experiment group: connected with SDZ-II electroacupuncture instrument (Suzhou Medical Appliance Factory, China; registration No. 20132270611) on the basis of the control group, using continuous wave, the frequency was 130∼260 times / min, current intensity is adjusted to the acceptable range of patients, generally at 1∼1.6 mA. Lasted for 30 min, 4 times a week for 4 weeks.

All patients are followed up for 30 days after treatment, and the curative effect is evaluated again through outpatient follow-up on the 15th day and the 30th day, and the score data are collected.

### 2.7 Evaluation criteria and efficacy judgment

(1) Primary outcome measures: Pain is the most important clinical manifestations of CSR patients, the VAS^[20]^ will be used to evaluate patients’ pain changes, and VAS is often used to measure pain intensity.^[22]^ Patients will be asked to mark a point between 0 and 100, where 100 represent the greatest pain (rightmost) and 0 indicates no pain (leftmost).
(2) Secondary Outcomes:①Neck Disability Index (NDI),^[23]^ which is one of the most commonly used indexes to evaluate the neck dysfunction of patients. The NDI score included 4 subjective symptoms (neck pain intensity, headache, attention, sleep) and 6 items related to activities of daily life (weightlifting, work, driving or cycling, entertainment, personal care, reading); ②Yasuhisa Tanaka 20 Score Scale,^[24]^ which is more specific to CSR, includes scores of sensation, muscle strength and other neurological functions in addition to pain.
(3) Adverse Reactions: Nausea, dizziness, rash, allergy and other discomforts occurred during the treatment.

### 2.8 Data collection and management

Two assistants collect and record the whole process of data during the study. Personal information about potential participants and registered participants will be collected, shared and stored in a separate storeroom to protect pre-trial, during and post-trial confidentiality. Access to the database will be limited to the researchers of this research team.

### 2.9 Statistical Analysis

The collected data are analyzed by SPSS 18.0 software. Chi-square test will be used for counting data, mean ± standard deviation () is used for measurement data, independent sample t test is used for normal distribution, Mann – Whitney *U* test is used for skewed distribution, and the difference was considered to be statistically significant when *P* *<* .05.

## 3 Discussion

With the popularity of mobile phones and computers and the increase of dependence on their use, the incidence of cervical spondylosis is also gradually increasing, and shows a younger trend.^[25]^ The risk factors of CSR include cervical degeneration, trauma, strain, cervical dysplasia, inflammation and so on. Its clinical feature is that the range of pain is consistent with that of the compressed spinal nerve area, which is mainly characterized by pain, numbness and hypoesthesia. In severe cases, muscle weakness and muscle atrophy may occur.

In China, electroacupuncture, like acupuncture, is widely used in the treatment of various diseases with definite efficacy and high safety.^[26]^ Electroacupuncture through a specific intensity and frequency of micro pulse current to strengthen the stimulation of acupoints, to enhance the effect of curative effect. Studies have found that electroacupuncture acts on acupoints and continuously stimulates the acupoints to promote the release of endogenous opioid peptides in the body.^[27]^ Endogenous opioid peptides can combine with opioid peptide receptors to produce analgesic effect.^[28]^ Experimental studies have found that electroacupuncture can inhibit Wnt- β-catenin signal pathway and slow down the degeneration of intervertebral disc.^[29]^ At present, some evidence shows that electroacupuncture seems to have an advantage over traditional acupuncture in the treatment of CSR. However, there are no rigorous clinical studies to directly compare the two schemes. Therefore, we intend to directly compare the efficacy of the two through prospective randomized controlled trials. The results of this study will be helpful to clinical practice and provide a basis for guiding patients with CSR to choose appropriate treatment.

The main limitation of this study is that due to the limitation of treatment, we are unable to achieve strict double blindness, which may affect the results.

## Author contributions

**Data curation:** Yazhou Zhou, WenGang Wang.

**Funding acquisition:** Yazhou Zhou.

**Resources:** WenGang Wang, Ke Tian.

**Software:** Mengrui Jia.

**Supervision:** Ke Tian, Hui Huang.

**Writing – original draft:** Yazhou Zhou, WenGang Wang.

**Writing – review & editing:** Yazhou Zhou.
